# Emergency surgeons’ perceptions and attitudes towards antibiotic prescribing and resistance: a worldwide cross-sectional survey

**DOI:** 10.1186/s13017-018-0190-5

**Published:** 2018-06-28

**Authors:** Francesco M. Labricciosa, Massimo Sartelli, Sofia Correia, Lilian M. Abbo, Milton Severo, Luca Ansaloni, Federico Coccolini, Carlos Alves, Renato Bessa Melo, Gian Luca Baiocchi, José-Artur Paiva, Fausto Catena, Ana Azevedo

**Affiliations:** 1 Department of Biomedical Science and Public Health, School of Hygiene and Preventive Medicine, Faculty of Medicine and Surgery, Università Politecnica delle Marche, Ancona, Italy; 2 Department of Surgery, Macerata Hospital, Macerata, Italy; 3 Epidemiology Research Unit (EPIUnit), Instituto de Saúde Pública, Universidade do Porto (ISPUP), Porto, Portugal; 4 Departamento de Ciências da Saúde Pública e Forenses e Educação Médica, Faculdade de Medicina, Universidade do Porto, Porto, Portugal; 5 Infection Prevention and Antimicrobial Stewardship Jackson Health System, University of Miami Miller School of Medicine, Miami, FL USA; 6 General Surgery Department, Papa Giovanni XXIII Hospital, Bergamo, Italy; 7 Unit of Prevention and Control of Infections and Antimicrobial Resistance (UPCIRA), Centro de Epidemiologia Hospitalar, Centro Hospitalar São João, Porto, Portugal; 8 Department of General Surgery, Centro Hospitalar São João, Porto, Portugal; 9 Department of Clinical and Experimental Sciences, University of Brescia, Brescia, Italy; 10 Department of Emergency and Intensive Care, Centro Hospitalar São João, Porto, Portugal; 11 Department of Medicine, Faculdade de Medicina, Universidade do Porto, Porto, Portugal; 12 Department of Emergency Surgery, Maggiore Hospital, Parma, Italy; 13 Centro de Epidemiologia Hospitalar, Centro Hospitalar São João, Porto, Portugal

**Keywords:** Cross-sectional survey, Emergency surgery, Antimicrobial stewardship, Antibiotic prescribing, Antibiotic resistance

## Abstract

**Background:**

Antibiotic resistance (AMR) is a growing public health problem worldwide, in part related to inadequate antibiotic use. A better knowledge of physicians’ motivations, attitudes and practice about AMR and prescribing should enable the design and implementation of effective antibiotic stewardship programs (ASPs). The objective of the study was to assess attitudes and perceptions concerning AMR and use of antibiotics among surgeons who regularly perform emergency or trauma surgery.

**Methods:**

A cross-sectional web-based survey was conducted contacting 4904 individuals belonging to a mailing list provided by the World Society of Emergency Surgery. Participation was voluntary and anonymous. The survey was open for 5 weeks (from May 3, 2017, to June 6, 2017), within which two reminders were sent. The self-administered questionnaire was developed by a multidisciplinary team; reliability and validity were assessed.

**Results:**

The overall response rate was 12.5%. Almost all participants considered AMR an important worldwide problem, but 45.6% of them underrated the problem in their own hospitals. Surgeons provided with periodic reports on local AMR demonstrated a lower underrating in their hospital. Only 66.3% of the surgeons stated to receive periodic reports on local AMR data, and among them, 56.2% had consulted them to select an antibiotic in the previous month. Availability of systematic reports about AMR, availability of guidelines for therapy of infections, and advice from an infectious diseases specialist were considered very helpful measures to improve antibiotic prescribing by 68.0, 65.7, and 64.9%, respectively. Persuasive and restrictive ASPs were both considered helpful measures by 64.5%. Moreover, 86.3% considered locally developed guidelines more useful than national ones. Only 21.9% received formal training in antibiotic prescribing in the previous year; among them, 86.6% declared to be interested in receiving more training.

**Conclusions:**

Availability of periodic reports on local AMR data was considered an important tool to guide surgeons in choosing the correct antibiotic and to increase awareness of the problem of AMR. Local guidelines for therapy of infections should be implemented in every emergency surgery setting, and developed by a multidisciplinary team directly involving surgeons, infectious diseases specialists, and microbiologists, and formally established in an ASP.

**Electronic supplementary material:**

The online version of this article (10.1186/s13017-018-0190-5) contains supplementary material, which is available to authorized users.

## Background

Antibiotic resistance (AMR) is a serious and growing public health problem in both hospital- and community-acquired infections worldwide [1], in part related to inadequate antibiotic use [2, 3]. Spreading of antibiotic-resistant bacteria has a negative impact on patient outcomes such as prolonged morbidity, hospital stay, and increased risk of death [4], resulting in increased health care costs and financial burden [5]. Development, namely through selection, of AMR is accelerated by inadequate antibiotic exposure [6]. Studies have estimated that between 20 and 50% of antibiotic use is either unnecessary or inappropriate and decreasing misuse is a necessary step of the strategy to curb antibiotic resistance [3, 7].

Antibiotics, unlike many other drugs, are utilized by virtually all doctors, across a wide spectrum of practices and various levels of training and knowledge [8–10]. In spite of the severe consequences and global spread of antibiotic resistance, effective dissemination of information to healthcare professionals about adverse outcomes associated with antibiotic misuse and assurance of an evidence-based approach in practice remain challenging [11].

Antimicrobial stewardship programs (ASPs) have emerged as a strategy to tackle the problem of AMR, as a systematic approach to improve and optimize the appropriate prescription of antibiotics through a variety of interventions and have been proven to be cost-effective [12]. ASPs should promote education, feedback, and effect changes in prescribing behaviors of healthcare providers [13]. In order to better plan these behavioral interventions, it is important to understand physicians’ motivation, knowledge, attitude, and practice [14]. A multidisciplinary collaboration among various specialties within a healthcare institution is essential to ensure that antibiotic management maximizes patient clinical outcomes and minimizes emergence and selection of AMR. In this context, the direct involvement of surgeons in ASPs can be highly impactful [15].

Emergency surgical admissions account for approximately half of all surgical admissions [16]. Emergency operative procedures are associated with an increased risk for surgical site infections (SSIs) [17], since they do not allow for the standard preoperative preparation normally performed for an elective operation. Typically performed on critical patients, emergency operative procedures are often carried out on contaminated or dirty wounds which are clearly identified as a significant risk factor for SSIs [18, 19]. Therefore, the role of the emergency surgeon is paramount in prescribing antibiotics judiciously, both for therapeutic use and preoperative prophylaxis. The necessity of systematic approaches for the optimization of antibiotic therapy in surgical units has become increasingly urgent [20].

Previous surveys have been conducted in hospital settings to assess physicians’ perceptions, attitudes, and knowledge about antibiotic use and resistance, including physicians from various specialties [13, 21–29]. Three surveys focused their investigation upon all physicians of targeted hospitals [30–32]. However, in these studies, mainly aggregated data were provided, without specifically analysing surgeons’ perceptions, attitudes, and knowledge apart from other professionals. Therefore, to the best of our knowledge, no previous physician surveys have focused only on surgeons, and they are one of the most frequent prescribers of broad spectrum antibiotics. Thus, it seems important to better understand the perceptions and attitudes of this group of prescribers worldwide.

We surveyed junior and senior surgeons, belonging to all surgical specialties, performing regularly emergency or trauma surgery in their activities. The objective of our study was to assess their knowledge, attitudes, and perceptions concerning antibiotic resistance and prescribing, in order to gain a deeper understanding of these processes in different cultural contexts, so as to provide information to enable the design and implementation of more effective antibiotic stewardship interventions in emergency and trauma surgery settings.

## Methods

We conducted a cross-sectional web-based survey evaluating emergency surgeons’ perceptions, attitudes, and knowledge about antibiotic use and resistance. The study was promoted by the World Society of Emergency Surgery (WSES) and by the Global Alliance for Infections in Surgery (GAIS) [33].

The population target was represented by the surgeons who regularly perform emergency or trauma surgery. Participants were registered as WSES ordinary members or professionals who subscribed to the newsletter of the World Journal of Emergency Surgery (WJES). A total of 4904 individuals were contacted via e-mail with an invitation letter and a survey link (http://www.docs.google.com), using a mailing list provided by the WSES. Although all members were invited to participate, only surveys completed by surgeons regularly performing emergency or trauma surgery were included in the analysis. The survey was written in English, participation was voluntary and anonymous, and no incentives for participation were given. The survey was opened for 5 weeks between May 3, 2017, and June 6, 2017. Two reminders were sent: the first one after 14 days and the second one after 28 days.

The self-administered questionnaire was developed by a multidisciplinary team of investigators including epidemiologists, surgeons, infectious diseases physicians, pharmacologists, and a statistician, after searching the medical literature for comparable studies and adapting questions designed in other physicians’ surveys previously carried out [34, 35]. The questionnaire (see Additional file 1) started with a characterization of the surgeons’ professional profiles (country, sex, surgical speciality, years of experience) and working setting (type of hospital, hospital inpatient beds, existence of an antimicrobial stewardship team, implementation of local guidelines for therapy of infections, and availability of periodic reports on local antibiotic resistance data). It included questions about surgeons’ perception regarding the importance and the causes of antibiotic resistance and attitudes towards antibiotic prescribing and about interventions designed to improve antimicrobial stewardship. Participants’ attitudes during the antibiotic prescribing process, perceptions of the factors influencing that process, and perceptions of the helpfulness of potential interventions to improve it were surveyed.

Published recommendations for the development and implementation of web-based surveys were applied to the design of our questionnaire [36, 37]. Questions about attitudes and perceptions towards antibiotic prescribing and resistance were designed using the 4-point Likert scale with response options from very helpful/important/confident to very unhelpful/unimportant/unconfident.

Anonymous data were automatically entered in an Excel database (Microsoft Corporation, Redmond, Washington, USA). The study was approved by the Ethics Committee of the Institute of Public Health of the University of Porto (ISPUP), which waived the need for written informed consent from the participants considering the anonymous nature of the collected data.

### Statistical analysis

To assess reliability and validity of the instrument, an online invitation letter was sent in May 2017 to 150 members randomly selected from the WSES mailing list, asking them to complete the questionnaire on a voluntary basis. Responses from 102 individuals were used to test content validity and reliability of the questionnaire (48 non-respondents). From those respondents, a sample of 31 individuals was used to test the reproducibility of the questionnaire.

We started assessing the dimensionality of the scale using principal component analysis with varimax rotation. The scree plot was used to define the number of dimensions, and the items whose factor loadings were greater than 0.4 were considered as being correlated with a specific principal component. The indirect reliability of the resulting domains was assessed using Cronbach’s alpha, in the overall sample. Test-retest reliability was assessed using consistency two-way mixed single intraclass correlation coefficient (ICC). We hypothesized that most of the subtests would have good test-retest reliability (minimum ICC of 0.70). Items were classified from 1 to 4. In each domain, the final score was estimated as the sum of the classification in the included items. Two independent-sample *t* test and analysis of variance (ANOVA) were used to compare the mean of final scores according to participants’ characteristics: sex, years of experience, type of hospital, availability of antimicrobial stewardship teams, local guidelines, and reports on AMR. In this process, statistical analysis was conducted using SPSS Statistical Package 21.0 (IBM Corporation, Armonk, NY, USA).

Descriptive analysis for categorical variables was presented in absolute frequency and percentage. Data obtained from questions concerning attitudes and perceptions and based on a 4-point Likert scale option were collapsed into two categories (very helpful/ important/confident and helpful/important/confident were collapsed into the first category; unhelpful/unimportant/unconfident and very unhelpful/unimportant/ unconfident were collapsed into the second category). The frequency of each category was compared by working setting and professional profile, existence of antimicrobial stewardship team, local guidelines, and reports using, as appropriate, chi-square or Fisher’s exact tests.

We defined “underrating” if the participant ranked (in a 4-point Likert scale option) the problem of AMR in its own institution less important than worldwide and “overrating” if the participant ranked (in a 4-point Likert scale option) its colleague’s prescriptions as more important contributing factor to AMR than its own.

All tests were two-sided; *p* values below 0.05 were considered statistically significant. Statistical calculations assessed on final data were performed using Stata 11 software package (StataCorp, College Station, TX, USA).

## Results

### Validity and reproducibility

Results are presented in Additional files 2, 3, 4, and 5. The principle component analysis (PCA) identified six principal components with eigenvalue higher than 1. The six principal components, hereafter referred to as domains, explained 58% of total variance. The Cronbach alpha was higher than 0.7 for domains 1, 2, 4, and 6, and only domains 3 and 5 had value lower than 0.7 (see Additional file 2). In the test-retest reliability analysis, the ICC ranged from 0.52 (score 1) to 0.82 (score 3) (see Additional file 3).

In the validation sample, we observed significant differences between sexes in scores 2 and 5, by years of experience in domain 2, and by type of hospital in domains 2 and 6 (see Additional file 4). In the final sample, we observed significant differences between sex in domains 1, 2, and 6; among years of experience in domain 4; among type of hospital in domains 2 and 6; and among local guidelines for therapy of infections in domains 2 and 5 (see Additional file 5).

### Baseline data: coverage, response rate, working setting, and professional profile

Six hundred thirty-seven of the 4904 professionals invited by e-mail to participate in the survey returned a filled-in questionnaire. Twenty-five participants stated they did not perform emergency or trauma surgery regularly; therefore, 612 questionnaires were deemed appropriate for analysis, with a final overall response rate of 12.5%. Surveyed emergency surgeons’ working setting and professional profile are described in Table 1.

**Table 1:** Surveyed surgeons’ working setting and professional profile

Characteristics	*N* (%)
WHO region classification	
Africa Region	35 (5.7)
Region of the Americas	128 (20.9)
South-East Asia Region	18 (2.9)
European Region	372 (60.8)
Easter Mediterranean Region	36 (5.9)
Western Pacific Region	23 (3.8)
Gender	
Male	526 (85.9)
Female	86 (14.1)
Surgeries regularly performed*	
Abdominal	577 (94.3)
Cardiac surgery	10 (1.6)
Gynecologic	30 (4.9)
Neurosurgery	4 (0.7)
Orthopedic	32 (5.2)
Pediatric	73 (11.9)
Thoracic	143 (23.4)
Urological	41 (6.7)
Vascular	74 (12.1)
Other	58 (9.5)
Years of experience	
Less than 10 years	126 (20.6)
10–20 years	210 (34.3)
21–30 years	155 (25.3)
More than 30 years	121 (19.8)
Type of hospital	
University hospital	395 (64.5)
Community teaching hospital	133 (21.7)
Community hospital	68 (11.1)
Other	16 (2.6)
Hospital inpatient beds	
Less than 100	27 (4.4)
100–500	225 (36.8)
501–1000	247 (40.4)
More than 1000	107 (17.5)
Unsure	6 (1.0)
Hospital with antimicrobial stewardship team	
Yes	448 (73.2)
No	139 (22.7)
Unsure	25 (4.1)
Local GLs for therapy of infections implemented	
Yes	465 (76.0)
No	137 (22.4)
Unsure	10 (1.6)
Reports on local AMR data periodically received	
Yes	406 (66.3)
No	177 (28.9)
Unsure	29 (4.7)

*WHO* World Health Organization, *GLs* guidelines, *AMR* antibiotic resistance

*Sum of numbers is greater than the overall sample (*n* = 612) since the question was based on a multiple choice

### Surgeons’ importance of the problem of antibiotic resistance and perceptions of causes of antibiotic resistance

Almost all the surveyed surgeons strongly agreed (493, 80.6%) or agreed (117, 19.1%) that antibiotic resistance is a worldwide problem; only two (0.3%) strongly disagreed. While the majority strongly agreed (240, 39.2%) or agreed (317, 51.8%), a minority disagreed (53, 8.7%) or strongly disagreed (2, 0.3%) that this is a problem in their own hospital. Two hundred seventy-nine (45.6%) surgeons underrated the problem in their hospital. Surgeons provided with periodic reports on local antibiotic resistance data underrated the problem in their hospitals less than their colleagues who did not receive these reports (39.9% versus 56.8%, *p* < 0.001).

**Fig. 1:**
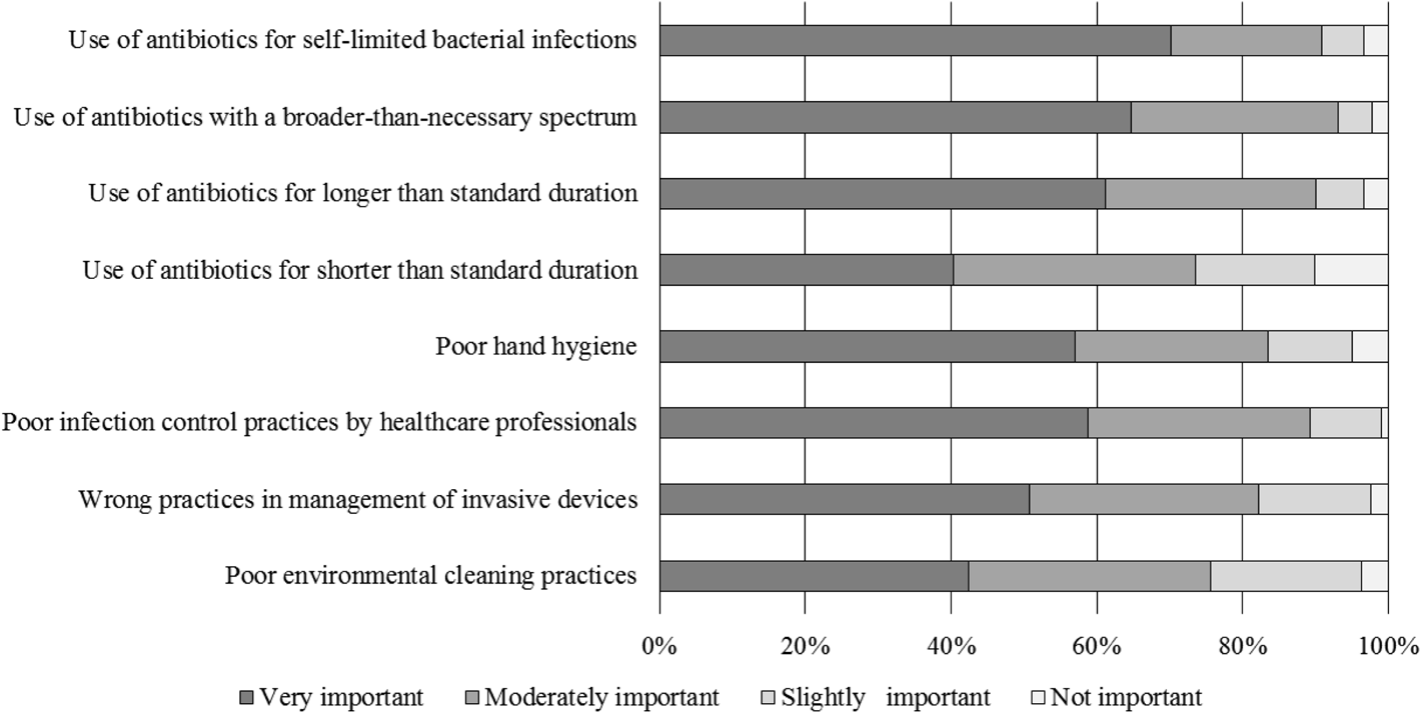
Perceptions of causes of antibiotic resistance

Perceptions of causes of AMR are reported in Fig. 1 and Table 2. Poor hand hygiene was more often considered an important cause of AMR by surgeons who declared to receive regularly report on resistance data than their colleagues who did not (85.7 versus 79.1%, *p* = 0.038). One hundred ninety-five (31.9%) surgeons overrated their colleagues’ prescriptions. However, we observed that surgeons whose surgical unit or department had local guidelines for therapy of infections tended to overrate less their colleagues’ prescriptions compared to surgeons who declared not to have them (28.2 versus 44.2%, *p* < 0.001). Overrating was similar throughout all other analysed characteristics.

**Table 2:** Perceptions of causes of antibiotic resistance

Questions	Very likely	Likely	Unlikely	Very unlikely
Do you think that your antibiotic prescriptions contribute to the problem of antibiotic resistance?	103 (16.8)	307 (50.2)	185 (30.2)	17 (2.8)
Do you think that your colleagues’ prescriptions contribute to the problem of antibiotic resistance?	155 (25.3)	381 (62.3)	72 (11.8)	4 (0.7)
Do you expect that antibiotic resistance will be a greater clinical problem for your patients in the future?	395 (64.5)	205 (33.5)	10 (1.6)	2 (0.3)
Do you expect that new antibiotics will be developed in the next 10 years will keep up with the problem of resistance?	88 (14.4)	230 (37.6)	262 (42.8)	32 (5.2)

### Participants’ attitudes during the antibiotic prescribing process

National guidelines for therapy of infections were used or consulted by 55.7% (341/612) of the surveyed surgeons, when considering an antibiotic for a patient in the previous month. Local guidelines for therapy of infections were used or consulted by 77.2% (359/465) of surgeons who stated to have them available in their surgical unit or department. Reports on local resistance data were personally consulted to define an antibiotic empiric therapy for a patient in the previous month by 56.2% (228/406) of the participants who declared to receive periodically these reports. No statistically significant differences in these three attitudes were found according to the working setting or professional profile.

**Fig. 2:**
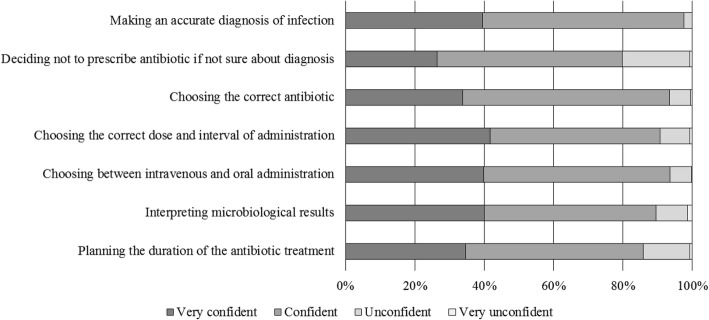
Confidence level for seven scenarios during an antibiotic prescribing process

Surveyed surgeons’ confidence levels for seven scenarios during an antibiotic prescribing process are described in Fig. 2. Surgeons working in hospitals where local guidelines for therapy of infections are implemented showed to be more confident in deciding not to prescribe an antibiotic if not sure about the diagnosis (82.6 versus 70.7%, *p* = 0.002) than those working in hospitals with no guidelines available. Moreover, those who stated to have consulted the guidelines in the previous month seemed to be more confident in choosing the correct antibiotic (93.9 versus 86.6%, *p* = 0.023).

### Perceptions of the factors influencing the antibiotic prescribing process

In the previous 12 months, 134 (21.9%) surgeons received formal training in antibiotic prescribing; among them, 116 (116/134, 86.6%) declared to be interested in receiving more training. On the contrary, 477 (77.9%) participants did not receive any training in antibiotic prescribing; among them, 370 (370/477, 77.6%) stated to be interested in receiving more training.

### Perceptions of the helpfulness of potential interventions to improve antibiotic prescribing

**Fig. 3:**
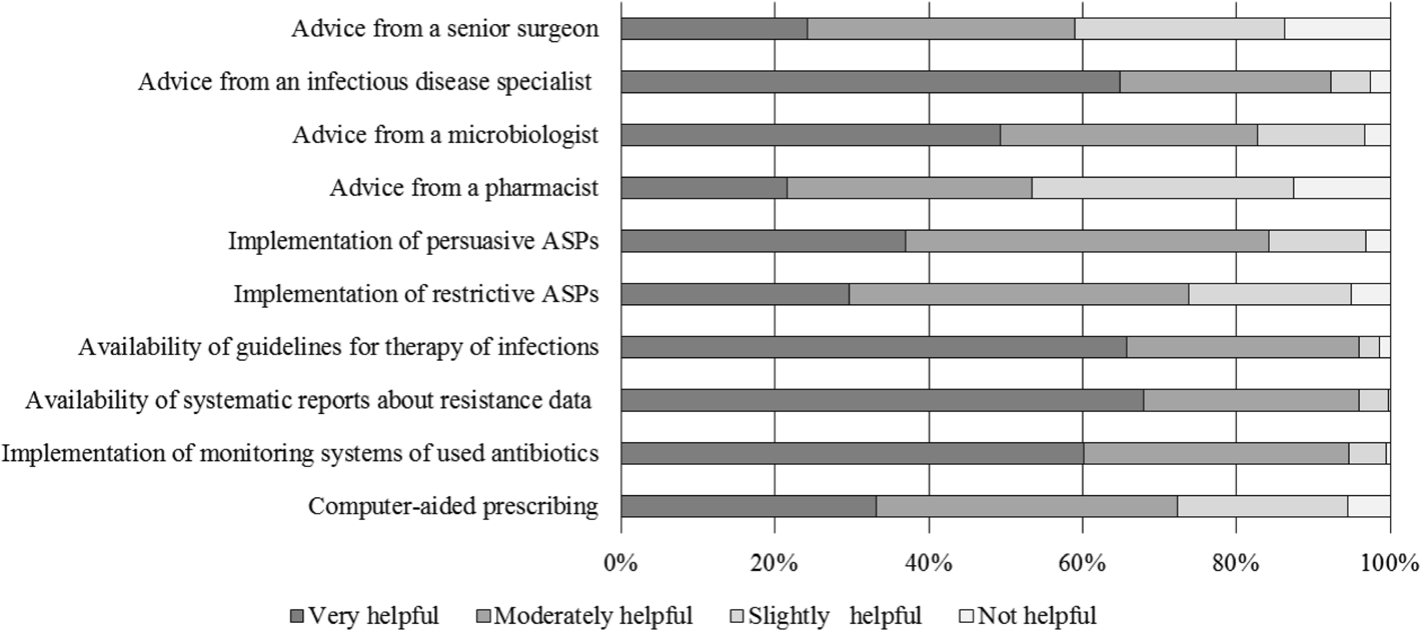
Surgeons’ ratings of the helpfulness of potential interventions to improve antibiotic prescribing

Surgeons’ ratings of the helpfulness of potential interventions to improve antibiotic prescribing are reported in Fig. 3. The majority of surveyed surgeons (395, 64.5%) attributed the same value to both persuasive and restrictive ASPs, while 145 (23.7%) considered persuasive ASPs more helpful than restrictive ASPs, and 72 (11.8%) found restrictive ASPs more helpful than persuasive ASP.

Participants who stated to receive periodically reports on resistant data rated this measure as very helpful (285/406, 70.2%) or helpful (107/406, 26.4%). Participants regularly receiving reports on local antibiotic resistance data considered helpful the implementation of restrictive measures more than their colleagues who did not receive them (76.8 versus 68.0%, *p* = 0.018). The majority of surveyed surgeons declared that locally developed guidelines for antibiotic treatment are more useful than national ones (528, 86.3%).

## Discussion

Our survey shows that surgeons of our sample are highly aware of and concerned about AMR, demonstrating awareness of a widespread issue that poses a threat for their patients. However, 45.6% of the surveyed surgeons underrated the problem in their own hospitals, perceiving the risk as more theoretical than real. These findings are consistent with other surveys previously published [21, 25, 28, 34]. However, it is noteworthy that surgeons provided with periodic reports on local antibiotic resistance data proved to have a lower underrating of the AMR issue in their hospital, probably demonstrating a better knowledge of local microbiology and consequently a higher awareness of the problem.

The availability of periodic reports on local rates of antibiotic resistance patterns is an essential component of the clinical decision-making process, since they can be used not only to evaluate trends of antibiotic resistance rates, but also to educate clinicians on optimal antibiotic use, and to assess the impact of interventions. Ideally, a microbiology service should provide analyses of AMR at least annually to both clinicians and antimicrobial stewardship committees, to inform local empirical therapy recommendations and formulary management [38]. This period should be adapted to every facility, taking into account human and financial resources required for its implementation and maintenance. Moreover, Infectious Diseases Society of America (IDSA) antimicrobial stewardship guidelines recommended to provide selective or cascade reporting to help guide appropriate antibiotic use [39], by taking into account both local susceptibility of the microorganisms and drug availability. In our study, just over half of the surveyed surgeons declaring to receive periodically reports on local resistance data had personally consulted them to select an antibiotic empiric therapy in the previous month. Even so, 70% of them stated that availability of reports on local resistance data is a very helpful measure to improve antibiotic prescribing. Therefore, in order to increase the active consultation of these reports, it is paramount to establish a solid communication between microbiologists and emergency surgeons, for example, through the existence of privileged interlocutors and the participation of microbiologists in regular surgeons’ meetings. This solution is likely to be welcomed by the emergency surgeons, since the vast majority of them considered helpful an advice from a microbiologist.

It is noteworthy that surgeons periodically provided with reports on local resistance data were more likely to consider poor hand hygiene an important cause of spread of AMR than their colleagues, highlighting again the importance of available reports in promoting the awareness of AMR. These findings should be emphasised, since both hand hygiene and infection control practices are effective preventive measures, stopping transmission of multidrug resistant organisms and preventing surgical site infections [40, 41]. Once multidrug-resistant organism infection or carriage is detected in hospitalized patients, in order to reduce person-to-person spread and prevent hospital diffusion, it is recommended the immediate implementation of standard and contact precautions [42]. Infection control measures have to be quickly implemented not only to minimize cross-transmission, but also to enable timely antimicrobial optimization, which, in turn, may lead to decreased deaths, shortened hospital stay, and lower hospitalization costs.

In our survey, only a minority of participants ranked restrictive ASPs more helpful than persuasive ones. Restrictive ASP may be perceived by the surveyed surgeons as a deprivation of autonomy in antibiotic prescribing [38], and its impact on surgeon autonomy may also create barriers to collaboration and communication with other members of the ASP [12]. Therefore, when restrictive interventions are required—as in urgent situations—it is important to add persuasive components to the programs since the first phases of its implementation.

As the majority of surveyed surgeons found the advice from an infectious diseases specialist very helpful and such strategy has recognised effectiveness in reducing antibiotic consumption and resistance with no impairment on clinical outcomes [43], a close collaboration between these two clinicians should be encouraged and formally included in the ASP, for example, implementing systematic bedside infectious disease consultation. A successful ASP should focus on collaboration among various professionals within a healthcare institution including prescribing clinicians. The quality of professional relationships between experts and non-experts remains a key component to achieving a real change and improvement.

Almost all the surveyed surgeons perceived locally developed treatment guidelines as a tool to improve antibiotic prescription. Furthermore, the majority declared that locally developed guidelines are more useful than national ones, as observed in a previous survey in a hospital setting [35]. However, almost one in four surgeons did not use or consult local guidelines when considering an antibiotic for a patient in the previous month. The attitude of relying on personal knowledge and experience rather than on recommendations of guidelines and formal policy was already reported and described by other authors [26, 28, 44]. In order to achieve optimal adherence to local antibiotic guidelines, more efforts are needed by antibiotic policy makers, promoting and achieving consensus before implementation and facilitating situations to make them more applicable.

Our study has strengths and limitations. To the best of our knowledge, no previous physician surveys have been focused only on emergency surgeons, in a worldwide perspective. Antibiotic prescription practices are affected by socio-cultural factors that vary across countries [33, 45], and we tried to address this issue extending the survey to surgeons from different countries all over the world. Moreover, the survey was conducted in a sample of surgeons with different working settings and professional profiles, supplying a wider framework in terms of surgical disciplines and years of experience. Furthermore, the study is methodologically robust, as the questionnaire underwent a formal statistical evaluation to ensure its validity and reliability. However, we selected the participants from a mailing list provided by a scientific society of emergency surgeons. Participants were not homogeneously distributed across all geographic regions of the world, and the majority of participants were from European countries, due to the difficulty in recruiting participants in some areas of the world. Participation rate was low which is in favor of some selection bias. However, participation might be underestimated because we were not able to define accurately the number of emergency or trauma surgeons among all the individuals invited to participate the survey. It is also possible that participation was more frequent among physicians with some interest or knowledge on the topic. Thus, generalizability may be impaired. Another potential limitation of any survey is the tendency of respondents to give socially desirable answers instead of revealing their true opinions. We tried to minimize this bias ensuring complete response anonymity with an online self-reported questionnaire.

## Conclusions

This study, conducted and focused on emergency surgeons, showed that availability of periodic reports on local rates of antibiotic resistance data should be considered an important tool to increase awareness of the problem of AMR. Prompt implementation of standard and contact precautions is an essential measure to stop transmission of multidrug-resistant organisms and prevent surgical site infections. Therefore, the active consultation of these reports should be encouraged and promoted through a dynamic collaboration between microbiologists and emergency surgeons, formally established in an ASP. Moreover, locally developed treatment guidelines should be implemented in every emergency surgery setting. They should be developed by a multidisciplinary team directly involving a surgeon, and efforts are needed to make them more applicable, and to achieve optimal adherence to them. In this context, the direct involvement of surgeons with knowledge in surgical infections in ASPs can be highly impactful, since they are at the forefront in treating patients with infections. Managers and antibiotic stewardship teams could take into account information from our survey in designing more targeted interventions in emergency surgery settings.

## Additional files

Additional file 1: Full-scale questionnaire. (DOCX 42 kb)

Additional file 2: Factor loadings from principal component analysis with varimax rotation and Cronbach alpha for each domain. (DOCX 18 kb)

Additional file 3: Test-retest of the domains: consistency two-way mixed single ICC. (DOCX 14 kb)

Additional file 4: Mean and standard deviation (SD) of each domain by surgeons’ professional profile and working setting (validation sample). (DOCX 19 kb)

Additional file 5: Mean and standard deviation (SD) of each domain by surgeons’ professional profile and working setting (final sample). (DOCX 18 kb)

## Abbreviations

ANOVA: Analysis of variance
AMR: Antibiotic resistance
ASPs: Antimicrobial stewardship programs
GAIS: Global Alliance for Infections in Surgery
ICC: Intraclass correlation coefficient
ISPUP: Institute of Public Health of the University of Porto
PC: Principle component
PCA: Principle component analysis
SD: Standard deviation
SSIs: Surgical site infections
WJES: World Journal of Emergency Surgery
WSES: World Society of Emergency Surgery

## References

[1] World Health Organization. In: Combat drug resistance. 2011. http://www.who.int/world-health-day/2011/en/. Accessed 14 Mar 2018.

[2] Goldman DA, Weinstein RA, Wenzel RP, Tablan OC, Duma RJ, Gaynes RP (1996). Strategies to prevent and control the emergent and spreads of antimicrobial resistant microorganisms in hospitals. JAMA.

[3] Dellit TH, Owens RC, McGowan JE, Gerding DN, Weinstein RA, Burke JP (2007). Infectious Diseases Society of America and the Society for Healthcare Epidemiology of America guidelines for developing an institutional program to enhance antimicrobial stewardship. Clin Infect Dis.

[4] Cosgrove SE (2006). The relationship between antimicrobial resistance and patient outcomes: mortality, length of hospital stay, and health care costs. Clin Infect Dis.

[5] Coast J, Smith R, Miller M (1996). Superbugs: should antimicrobial resistance be included as a cost in economic evaluation?. Health Econ.

[6] World Health Organization. In: Global strategy for the containment of antimicrobial resistance. 2001. http://www.who.int/drugresistance/WHO_Global_Strategy_English.pdf. Accessed 14 Mar 2018.

[7] Pulcini C, Cua E, Lieutier F, Landraud L, Dellamonica P, Roger PM (2007). Antibiotic misuse: a prospective clinical audit in a French university hospital. Eur J Clin Microbiol Infect Dis.

[8] Roumie CL, Halasa NB, Edwards KM, Zhu Y, Dittus RS, Griffin MR (2005). Differences in antibiotic prescribing among physicians, residents, and nonphysician clinicians. Am J Med.

[9] Running A, Kipp C, Mercer V (2006). Prescriptive patterns of nurse practitioners and physicians. J Am Acad Nurse Pract.

[10] Edgar T, Boyd SD, Palame MJ (2009). Sustainability for behaviour change in the fight against antibiotic resistance: a social marketing framework. J Antimicrob Chemother.

[11] Charani E, Cooke J, Holmes A (2010). Antibiotic stewardship programmes—what’s missing?. J Antimicrob Chemother.

[12] Davey P, Brown E, Fenelon L, Finch R, Gould I, Hartman G, et al. Interventions to improve antibiotic prescribing practices for hospital inpatients. Cochrane Database Syst Rev. 2005;(4):CD003543. 10.1002/14651858.CD003543.pub2 16235326

[13] Abbo L, Sinkowitz-Cochran R, Smith L, Ariza-Heredia E, Gómez-Marín O, Srinivasan A (2011). Faculty and resident physicians’ attitudes, perceptions and knowledge about antimicrobial use and resistance. Infect Control Hosp Epidemiol.

[14] Cabana MD, Rand CS, Powe NR, Wu AW, Wilson MH, Abboud PA (1999). Why don’t physicians follow clinical practice guidelines? A framework for improvement. JAMA.

[15] Cakmakci M (2015). Antibiotic stewardship programmes and the surgeon’s role. J Hosp Infect.

[16] Mai-Phan TA, Patel B, Walsh M, Abraham AT, Kocher HM (2008). Emergency room surgical workload in an inner city UK teaching hospital. World J Emerg Surg.

[17] Malone DL, Genuit T, Tracy JK, Gannon C, Napolitano LM (2002). Surgical site infections: reanalysis of risk factors. J Surg Res.

[18] Cruse PJ, Foord R (1980). The epidemiology of wound infection. A 10-year prospective study of 62,939 wounds. Surg Clin North Am.

[19] Culver DH, Horan TC, Gaynes RP, Martone WJ, Jarvis WR, Emori TG (1991). Surgical wound infection rates by wound class, operative procedure, and patient risk index. National Nosocomial Infections Surveillance System. Am J Med.

[20] Sartelli M, Duane TM, Catena F, Tessier JM, Coccolini F, Kao LS (2016). Antimicrobial stewardship: a call to action for surgeons. Surg Infect.

[21] Wester CW, Durairaj L, Evans AT, Schwartz DN, Husain S, Martinez E (2002). Antibiotic resistance: a survey of physician perceptions. Arch Intern Med.

[22] Guerra CM, Pereira CA, Neves Neto AR, Cardo DM, Correa L (2007). Physicians’ perceptions, beliefs, attitudes and knowledge concerning antimicrobial resistance in a Brazilian teaching hospital. Infect Control Hosp Epidemiol.

[23] Tennant I, Nicholson A, Gordon-Strachan GM, Thoms C, Chin V, Didier MA (2010). A survey of physicians’ knowledge and attitudes regarding antimicrobial resistance and antibiotic prescribing practices at the University Hospital of the West Indies. West Indian Med J.

[24] Garcia C, Llamocca LP, Garcia K, Jimenez A, Samalvides F, Gotuzzo E (2011). Knowledge, attitudes and practice survey about antimicrobial resistance and prescribing among physicians in a hospital setting in Lima, Peru. BMC Clin Pharmacol.

[25] Navarro-San Francisco C, Del Toro MD, Cobo J, De Gea-García JH, Vañó-Galván S, Moreno-Ramos F (2013). Knowledge and perceptions of junior and senior Spanish resident doctors about antibiotic use and resistance: results of a multicenter survey. Enferm Infecc Microbiol Clin.

[26] Thriemer K, Katuala Y, Batoko B, Alworonga JP, Devlier H, VanGeet C (2013). Antibiotic prescribing in DR Congo: a knowledge. Attitude and practice survey among medical doctors and students. PLoS One.

[27] Abera B, Kibret M, Mulu W (2014). Knowledge and beliefs on antimicrobial resistance among physicians and nurses in hospitals in Amhara Region, Ethiopia. BMC Pharmacol Toxicol.

[28] Baadani AM, Baig K, Alfahad WA, Aldalbahi S, Omrani AS (2015). Physicians’ knowledge, perceptions, and attitudes toward antimicrobial prescribing in Riyadh, Saudi Arabia. Saudi Med J.

[29] Alothman A, Algwizani A, Alsulaiman M, Alalwan A, Binsalih S, Knowledge BM (2016). Attitude of physicians toward prescribing antibiotics and the risk of resistance in two reference hospitals. Infect Dis (Auckl).

[30] Srinivasan A, Song X, Richard A, Sinkowitz-Cochran R, Cardo D, Rand C. A survey of knowledge, attitudes, and beliefs of house staff physicians from various specialties concerning antimicrobial use and resistance. Arch Intern Med 2004;164:1451–1456. 10.1001/archinte.164.13.1451 15249355

[31] Lucet JC, Nicolas-Chanoine MH, Roy C, Riveros-Palacios O, Diamantis S, Le Grand J (2011). Antibiotic use: knowledge and perceptions in two university hospitals. J Antimicrob Chemother.

[32] Kheder SI (2013). Physcians knowledge and perception of antimicrobial resistance: a survey in Khartoum Stata Hospital settings. Br J Pharmaceut Res.

[33] Global Alliance for Infections in Surgery. https://infectionsinsurgery.org. Accessed 3 May 2018.

[34] Pulcini C, Williams F, Molinari N, Davey P, Nathwani D (2011). Junior doctors’ knowledge and perceptions of antibiotic resistance and prescribing: a survey in France and Scotland. Clin Microbiol Infect.

[35] Abbo LM, Cosgrove SE, Pottinger PS, Pereyra M, Sinkowitz-Cochran R, Srinivasan A (2013). Medical students’ perceptions and knowledge about antimicrobial stewardship: how are we educating our future prescribers?. Clin Infect Dis.

[36] Eysenbach G, Wyatt J (2002). Using the Internet for surveys and health research. J Med Internet Res.

[37] Kelley K, Clark B, Brown V, Sitzia J (2003). Good practice in the conduct and reporting of survey research. Int J Qual Health Care.

[38] MacDougall C, Polk RE (2005). Antimicrobial stewardship programs in health care systems. Clin Microbiol Rev.

[39] Barlam TF, Cosgrove SE, Abbo LM, MacDougall C, Schuetz AN, Septimus EJ (2016). Implementing an Antibiotic Stewardship Program: guidelines by the Infectious Diseases Society of America and the Society for Healthcare Epidemiology of America. Clin Infect Dis.

[40] Siegel JD, Rhinehart E, Jackson M, Chiarello L, Health Care Infection Control Practices Advisory Committee (2007). 2007 guideline for isolation precautions: preventing transmission of infectious agents in health care settings. Am J Infect Control.

[41] Siegel JD, Rhinehart E, Jackson M, Chiarello L; Healthcare Infection Control Practices Advisory Committee. Management of multidrug-resistant organisms in health care settings, 2006. Am J Infect Control 2007;35:S165–S193. 10.1016/j.ajic.2007.10.006 18068814

[42] Tacconelli E, Cataldo MA, Dancer SJ, De Angelis G, Falcone M, Frank U (2014). ESCMID guidelines for the management of the infection control measures to reduce transmission of multidrug-resistant Gram-negative bacteria in hospitalized patients. Clin Microbiol Infect.

[43] Tedeschi S, Trapani F, Giannella M, Cristini F, Tumietto F, Bartoletti M (2017). An antimicrobial stewardship program based on systematic infectious disease consultation in a rehabilitation facility. Infect Control Hosp Epidemiol.

[44] Charani E, Castro-Sánchez E, Holmes A (2014). The role of behavior change in antimicrobial stewardship. Infect Dis Clin N Am.

[45] Harbarth S, Albrich W, Brun-Buisson C (2002). Outpatient antibiotic use and prevalence of antibiotic-resistant pneumococci in France and Germany: a sociocultural perspective. Emerg Infect Dis.

