# Supplementary material for: Emergency surgeons’ perceptions and attitudes towards antibiotic prescribing and resistance: a worldwide cross-sectional survey
Source: World J Emerg Surg. 2018 Jun 28;13:27. doi: 10.1186/s13017-018-0190-5 (PMC6027784; doi:10.1186/s13017-018-0190-5)
Supplement: Supplementary file 1 — Full-scale questionnaire. (DOCX 42 kb) [file 13017_2018_190_MOESM1_ESM.docx]

**Additional File 1. Full-scale questionnaire.**

*This questionnaire is addressed at surgeons who regularly perform emergency or trauma surgery in their activities.*

*The estimated time to answer the questionnaire is 10 minutes.*

**1. Do you regularly perform emergency or trauma surgery in your duties?** Yes No

_1_ _2_

*If yes, please continue.*

*If no, thanks for your collaboration.*

*Working setting and professional profile*

**2. In which country are you currently working?**

Please, choose from the list.

**3. Please, tell us your gender.**

Male _1_

Female _2_

**4. Which of the following surgeries do you regularly perform as part of your duties?**

Multiple choice is allowed: select all that apply.

Abdominal _1_

Gynaecologic _2_

Cardiac surgery _3_

Neurosurgery _4_

Orthopaedic _5_

Paediatric _6_

Thoracic _7_

Urological _8_

Vascular _9_

Other ______________ _10_

**5. Including residency training, how many years of experience do you have as a surgeon?**

Less than 10 years _1_

10-20 years _2_

21-30 years _3_

More than 30 years _4_

**6. What type of hospital are you working in? If you are working in more than one hospital, please take into account the one where you practice the most of your time.**

University Hospital _1_

Community Hospital _2_

Community teaching Hospital _3_

Other __________________ _4_

**7. How many inpatient beds has the hospital where you practice the most of your time?**

Less than 100 _1_

100-500 _2_

501-1000 _3_

More than 1000 _4_

Unsure _5_

**8. Does your hospital have an antimicrobial stewardship team?**

Yes No Unsure

_1_ _2_ _3_

**9. Does your Surgical Unit/Department have local guidelines for therapy of infections?**

Yes No Unsure

_1_ _2_ _3_

**10. Does your Surgical Unit/Department periodically receive reports on local antibiotic resistance data?**

Yes No Unsure

_1_ _2_ _3_

*Participants’ perceptions of the importance of the problem of antibiotic resistance and of causes of antibiotic resistance*

**11. Do you think that antibiotic resistance is a worldwide problem?**

Strongly agree Agree Disagree Strongly disagree

_1_ _2_  _3_ _4_

**12. Do you think that antibiotic resistance is a problem in your hospital?**

Strongly agree Agree Disagree Strongly disagree

_1_ _2_  _3_ _4_

**13. Tell us your perceptions for each of the following statements regarding their relevance as contributing factors to the development or spread of antimicrobial resistance:**

|  | Very important | Moderately important | Slightly important | Not important |  |
| --- | --- | --- | --- | --- | --- |
| 1. Use of antibiotics for self-limited non bacterial infections | _1_ | _2_ | _3_ | _4_ |  |
| 1. Use of antibiotics with a broader-than-necessary spectrum | _1_ | _2_ | _3_ | _4_ |  |
| 1. Use of antibiotics for longer than standard duration | _1_ | _2_ | _3_ | _4_ |  |
| 1. Use of antibiotics for shorter than standard duration | _1_ | _2_ | _3_ | _4_ |  |
| 1. Poor hand hygiene | _1_ | _2_ | _3_ | _4_ |  |
| 1. Poor infection control practices by healthcare professionals | _1_ | _2_ | _3_ | _4_ |  |
| 1. Wrong practices in management of invasive devices | _1_ | _2_ | _3_ | _4_ |  |
| 1. Poor environmental cleaning practices | _1_ | _2_ | _3_ | _4_ |  |

**14. Do you think that your antibiotic prescriptions contribute to the problem of antibiotic resistance?**

Highly likely Likely Unlikely Highly unlikely

_1_ _2_  _3_ _4_

**15. Do you think that your colleagues’ prescriptions contribute to the problem of antibiotic resistance?**

Highly likely Likely Unlikely Highly unlikely

_1_ _2_  _3_ _4_

**16. Do you expect that antibiotic resistance will be a greater clinical problem for your patients in the future?**

Highly likely Likely Unlikely Highly unlikely

_1_ _2_  _3_ _4_

**17. Do you expect that new antibiotics will be developed in the next 10 years will keep up with the problem of resistance?**

Highly likely Likely Unlikely Highly unlikely

_1_ _2_  _3_ _4_

*Participants’ attitudes during the antibiotic prescribing process, perceptions of the factors influencing the antibiotic prescribing process and perceptions of the helpfulness of potential interventions to improve antibiotic prescribing*

**18. In the last month, have you personally used or consulted local guidelines for therapy of infections when considering an antibiotic for a patient?**

Please, select “Not applicable” if you did not prescribe any antibiotic in the last month or if your Surgical Unit/Department has no local guidelines for therapy of infections.

Yes No Unsure Not applicable _1_ _2_ _3_ _4_

**19. In the last month, have you personally used or consulted national guidelines for therapy of infections when considering an antibiotic for a patient?**

Please, select “Not applicable” if you did not prescribe any antibiotic in the last month.

Yes No Unsure Not applicable _1_ _2_ _3_ _4_

**20. In the last month, have you personally consulted reports on local resistance data to select an antibiotic empiric therapy for a patient?**

Please, select “Not applicable” if your hospital does not provide you any resistance data report.

Yes No Unsure Not applicable _1_ _2_ _3_ _4_

**21. Considering your daily clinical activities at the moment, how confident do you feel in the following scenarios when prescribing an antibiotic?**

|  | Very confident | Confident | Unconfident | Very Unconfident |
| --- | --- | --- | --- | --- |
| 1. Making an accurate diagnosis of infection | _1_ | _2_ | _3_ | _4_ |
| 1. Deciding not to prescribe an antibiotic if you are not sure about your diagnosis | _1_ | _2_ | _3_ | _4_ |
| 1. Choosing the correct antibiotic | _1_ | _2_ | _3_ | _4_ |
| 1. Choosing the correct dose and interval of administration | _1_ | _2_ | _3_ | _4_ |
| 1. Choosing between intravenous and oral administration | _1_ | _2_ | _3_ | _4_ |
| 1. Interpreting microbiological results | _1_ | _2_ | _3_ | _4_ |
| 1. Planning the duration of the antibiotic treatment | _1_ | _2_ | _3_ | _4_ |

**22. In the last 12 months, have you received formal** **training in antibiotic prescribing?**

Yes No Unsure

_1_ _2_ _3_

**23. Would you like to receive more training in antibiotic prescribing in your hospital?**

Yes No Unsure

_1_ _2_ _3_

**24. Please evaluate, in your opinion, the helpfulness of the following measures to improve antibiotic prescribing at the moment:**

|  | Very helpful | Moderately helpful | Slightly helpful | Not helpful |
| --- | --- | --- | --- | --- |
| 1. Advice from a senior surgeon | _1_ | _2_ | _3_ | _4_ |
| 1. Advice from an infectious disease specialist | _1_ | _2_ | _3_ | _4_ |
| 1. Advice from a microbiologist | _1_ | _2_ | _3_ | _4_ |
| 1. Advice from a pharmacist | _1_ | _2_ | _3_ | _4_ |
| 1. Implementation of persuasive ASPs | _1_ | _2_ | _3_ | _4_ |
| 1. Implementation of restrictive ASPs | _1_ | _2_ | _3_ | _4_ |
| 1. Availability of locally developed guidelines for therapy of infections | _1_ | _2_ | _3_ | _4_ |
| 1. Availability of systematic reports about resistance data | _1_ | _2_ | _3_ | _4_ |
| 1. Implementation of monitoring systems of used antibiotics | _1_ | _2_ | _3_ | _4_ |
| 1. Computer-aided prescribing | _1_ | _2_ | _3_ | _4_ |

**25. Do you think locally developed guidelines for antibiotic treatment are more useful than national ones?**

Strongly agree Agree Disagree Strongly disagree

_1_ _2_  _3_ _4_
