# Supplementary material for: Emergency surgeons’ perceptions and attitudes towards antibiotic prescribing and resistance: a worldwide cross-sectional survey
Source: World J Emerg Surg. 2018 Jun 28;13:27. doi: 10.1186/s13017-018-0190-5 (PMC6027784; doi:10.1186/s13017-018-0190-5)
Supplement: Supplementary file 2 — Factor loadings from principal component analysis with varimax rotation and Cronbach alpha for each domain. (DOCX 18 kb) [file 13017_2018_190_MOESM2_ESM.docx]

**Additional File 2. Factor loadings from principal components analysis with varimax rotation and Cronbach alpha for each domain.**

| **Domains and questions** | **PCA1** | **PCA2** | **PCA3** | **PCA4** | **PCA5** | **PCA6** | **Cronbach alpha** |
| --- | --- | --- | --- | --- | --- | --- | --- |
| **Domain 1 ^a^**  **Question 13a**  **Question 13b**  **Question 13c** | 0.06  0.12  -0.09 | 0.08  0.20  0.10 | **0.67**  **0.76**  **0.81** | -0.05  0.20  0.03 | 0.13  -0.13  0.22 | -0.08  0.14  0.15 | 0.73 |
| **Domain 2 ^b^**  **Question 13d**  **Question 13e**  **Question 13f**  **Question 13g**  **Question 13h** | **0.42**  **0.82**  **0.83**  **0.76**  **0.75** | -0.04  -0.08  0.15  0.09  0.16 | 0.11  0.04  0.08  -0.04  -0.09 | 0.13  0.15  0.06  0.07  0.02 | 0.21  -0.01  0.16  0.26  0.32 | -0.28  -0.03  0.00  0.16  0.06 | 0.82 |
| **Domain 3 ^c^**  **Question 14**  **Question 15**  **Question 16** | -0.08  0.19  -0.09 | -0.13  0.00  -0.28 | -0.04  0.10  0.26 | -0.02  0.02  0.11 | 0.00  0.08  0.00 | **0.66**  **0.77**  **0.39** | 0.50 |
| **Domain 4 ^d^**  **Question 21a**  **Question 21b**  **Question 21c**  **Question 21d**  **Question 21e**  **Question 21f**  **Question 21g** | 0.25  0.24  0.07  0.18  -0.10  -0.11  -0.11 | **0.57**  **0.59**  **0.74**  **0.80**  **0.76**  **0.62**  **0.72** | 0.33  -0.16  0.23  -0.03  0.09  0.03  0.04 | -0.15  0.25  0.07  0.06  0.05  -0.24  -0.03 | 0.11  -0.03  0.04  -0.07  0.23  0.36  0.24 | 0.05  0.16  -0.08  -0.05  -0.09  -0.16  -0.15 | 0.84 |
| **Domain 5 ^e^**  **Question 24a**  **Question 24b**  **Question 24c**  **Question 24d**  **Question 24l** | 0.11  0.36  0.15  -0.06  0.01 | 0.21  0.02  0.03  0.07  -0.18 | -0.45  0.04  -0.07  0.08  0.11 | **0.46**  **0.64**  **0.72**  **0.76**  **0.51** | 0.00  -0.12  0.25  0.12  0.27 | 0.11  -0.15  -0.22  0.14  0.15 | 0.67 |
| **Domain 6 ^f^**  **Question 24e**  **Question 24f**  **Question 24g**  **Question 24h**  **Question 24i** | 0.17  0.20  0.18  0.15  0.23 | 0.12  -0.01  0.15  0.20  0.12 | 0.11  -0.06  0.02  0.10  0.13 | 0.12  0.39  0.16  -0.10  0.25 | **0.62**  **0.50**  **0.56**  **0.71**  **0.66** | 0.24  0.25  0.10  -0.20  -0.21 | 0.74 |
| **Explained variance**  **PV**  **CV** | 12%  25% | 13%  13% | 8%  51% | 9%  43% | 9%  34% | 6%  58% |  |

PCA: Principle Component Analysis. PV: Proportion Variance Explained. CM: Cumulative Explained Variance. ^a^ Relevance as contributing factors to the development or spread of AMR: use of antibiotics. ^b^ Relevance as contributing factors to the development or spread of AMR: infection control measures. ^c^ Factors contributing to the spread of AMR. ^d^ Confidence in prescribing antibiotics. ^e^ Helpfulness of advice or computer-aided. ^f^ Helpfulness of implementation of antimicrobial stewardship measures.
