# Supplementary material for: Emergency surgeons’ perceptions and attitudes towards antibiotic prescribing and resistance: a worldwide cross-sectional survey
Source: World J Emerg Surg. 2018 Jun 28;13:27. doi: 10.1186/s13017-018-0190-5 (PMC6027784; doi:10.1186/s13017-018-0190-5)
Supplement: Supplementary file 4 — Mean and standard deviation (SD) of each domain by surgeons’ professional profile and working setting (validation sample). (DOCX 19 kb) [file 13017_2018_190_MOESM4_ESM.docx]

**Additional File 4. Mean and standard deviation (SD) of each domain by surgeons’ professional profile and working setting (validation sample).**

| **Variables** | **Domain 1 ^a^**  **mean (SD)** | **Domain 2 ^b^**  **mean (SD)** | **Domain 3 ^c^**  **mean (SD)** | **Domain 4 ^d^**  **mean (SD)** | **Domain 5 ^e^**  **mean (SD)** | **Domain 6 ^f^**  **mean (SD)** |
| --- | --- | --- | --- | --- | --- | --- |
| **Sex**  **Male**  **Female**  **P value** | 4.22 (1.66)  3.69 (1.18)  0.168 | **8.94 (3.34)**  **7.15 (1.63)**  **0.004** | 12.67 (3.20)  10.92 (3.01)  0.070 | 9.98 (2.72)  8.77 (2.13)  0.082 | **8.38 (2.42)**  **6.92 (1.65)**  **0.011** | 5.36 (1.27)  5.77 (2.13)  0.510 |
| **Years of experience**  **Less than 10 years**  **11 - 20 years**  **21 - 30 years**  **More than 30 years**  **P value** | 4.33 (2.01)  4.28 (1.75)  4.00 (0.95)  3.71 (1.20)  0.625 | **7.79 (2.36)**  **8.74 (2.92)**  **7.33 (2.44)**  **12.29 (3.99)**  **<0.001** | 12.92 (3.06)  12.60 (3.16)  12.05 (3.83)  11.79 (2.78)  0.681 | 9.25 (2.49)  10.21 (2.40)  9.48 (3.70)  10.14 (1.96)  0.473 | 8.83 (2.58)  8.09 (2.16)  7.24 (1.87)  8.86 (3.01)  0.100 | 5.50 (1.48)  5.42 (1.30)  5.10 (1.38)  5.71 (1.38)  0.620 |
| **Type of hospital**  **University hospital**  **Community teaching hospital**  **Community hospital**  **P value** | 4.25 (1.58)  4.11 (2.14)  3.90 (0.74)  0.566 | **9.54 (3.32)**  **6.94 (1.92)**  **6.70 (2.16)**  **0.001** | 12.58 (3.22)  12.83 (3.22)  11.00 (2.91)  0.488 | 9.86 (2.80)  9.00 (2.38)  11.30 (1.70)  0.169 | 8.28 (2.58)  8.22 (2.18)  7.30 (1.25)  0.609 | **5.41 (1.35)**  **4.78 (1.40)**  **6.60 (1.17)**  **0.011** |
| **Hospital with antimicrobial stewardship team**  **Yes**  **No**  **Unsure**  **P value** | 4.13 (1.55)  4.30 (1.86)  3.80 (1.30)  0.795 | 8.83 (3.48)  8.15 (2.63)  10.20 (1.92)  0.375 | 12.33 (3.38)  12.33 (2.83)  14.80 (2.17)  0.248 | 9.69 (2.62)  9.89 (2.95)  11.40 (1.67)  0.384 | 8.07 (2.49)  8.22 (2.15)  9.80 (1.64)  0.294 | 5.39 (1.46)  5.41 (1.28)  5.80 (1.48)  0.819 |
| **Local guidelines for therapy of infections**  **Yes**  **No**  **Unsure**  **P value** | 4.16 (1.77)  4.15 (1.12)  4.10 (1.37)  0.993 | 8.68 (3.37)  9.10 (3.23)  8.20 (2.10)  0.767 | 12.58 (3.13)  12.05 (3.73)  12.30 (3.09)  0.813 | 9.86 (2.34)  10.05 (3.79)  9.10 (2.72)  0.647 | 8.19 (2.36)  7.89 (2.60)  8.80 (2.20)  0.627 | 5.51 (1.51)  5.26 (1.19)  5.00 (1.82)  0.498 |
| **Reports on local antibiotic resistance data**  **Yes**  **No**  **Unsure**  **P value** | 4.03 (1.40)  4.44 (2.15)  4.44 (1.67)  0.499 | 8.89 (3.45)  8.26 (2.83)  8.56 (2.46)  0.718 | 12.43 (3.19)  11.65 (3.08)  14.67 (3.04)  0.056 | 9.76 (2.78)  9.65 (2.17)  10.78 (3.15)  0.532 | 7.93 (2.22)  8.83 (2.59)  8.67 (2.87)  0.243 | 5.41 (1.56)  5.43 (0.95)  5.33 (1.22)  0.983 |

SD: standard deviation. ^a^ Relevance as contributing factors to the development or spread of AMR: use of antibiotics. ^b^ Relevance as contributing factors to the development or spread of AMR: infection control measures. ^c^ Factors contributing to the spread of AMR. ^d^ Confidence in prescribing antibiotics. ^e^ Helpfulness of advice or computer-aided. ^f^ Helpfulness of implementation of antimicrobial stewardship measures.
